# An Atlas of the Speed of Copy Number Changes in Animal Gene Families and Its Implications

**DOI:** 10.1371/journal.pone.0007342

**Published:** 2009-10-23

**Authors:** Deng Pan, Liqing Zhang

**Affiliations:** 1 Department of Computer Science, Virginia Tech, Blacksburg, Virginia, United States of America; 2 Program in Genetics, Bioinformatics, and Computational Biology, Blacksburg, Virginia, United States of America; University of Texas Arlington, United States of America

## Abstract

The notion that gene duplications generating new genes and functions is commonly accepted in evolutionary biology. However, this assumption is more speculative from theory rather than well proven in genome-wide studies. Here, we generated an atlas of the rate of copy number changes (CNCs) in all the gene families of ten animal genomes. We grouped the gene families with similar CNC dynamics into rate pattern groups (RPGs) and annotated their function using a novel bottom-up approach. By comparing CNC rate patterns, we showed that most of the species-specific CNC rates groups are formed by gene duplication rather than gene loss, and most of the changes in rates of CNCs may be the result of adaptive evolution. We also found that the functions of many RPGs match their biological significance well. Our work confirmed the role of gene duplication in generating novel phenotypes, and the results can serve as a guide for researchers to connect the phenotypic features to certain gene duplications.

## Introduction

Understanding how phenotypes are connected to genotypes is one of the core challenges for biologists. With the increasing number of sequenced genomes, many genome-wide comparative studies have emerged to explore this topic [1], [2], [3], [4], [5], [6], [7], [8], [9], [10]. As a general routine, these studies tried to use some features of the genome as measurements to compare between species and associate them with functional categories. For example, Lopez-Bigas et al.[10] used protein divergence and Vogel and Chothia[8] used organismic complexity to compare genomes of different species.

Since phenotypic difference is likely to be caused by new genes and functions, and gene duplication is believed to be one of the major mechanisms for organisms to generate new genes and functions [11], the dynamics of gene duplication can be a good feature for genome-wide comparisons between species. Recently, the tempo and mode of copy number changes (CNCs) in gene families, especially lineage-specific CNCs, has gained a lot of attention [1], [2], [3], [4], [6], [7], [5]. CNCs can be achieved by either gene gain or gene loss. In the crown-group eukaryotes, most of the gene families involved in lineage-specific expansions are shown to perform organizational and regulatory functions [2]. Meanwhile, the rate of gene loss in vertebrates seems to be much lower than that in protostomes, implying that vertebrate genomes tend to keep more complexity than those of “simpler” species [12], [13], [14]. However, there are also many gene families existing only in the “simpler” species but not in vertebrates [4]. In mammals, more than half of the gene families that are descended from the common ancestor of mammals have either gene gain or gene loss in at least one lineage [5], suggesting that gene duplication might have played an important role in the diversification of mammals.

To further understand this topic, we systematically investigated the rate of CNCs in ten animals and connected the variation in rates of CNCs with different functional groups. In contrast to previous studies, we measured the CNC rate using humans as the reference point to calibrate the speed. Our method does not need to assume any predefined gene duplication model [4] or build phylogenetic trees for gene families in all species [7], both of which are error-prone. Due to complex situations during the fixation of duplicated genes, no single quantitative model is satisfactory to account for the duplication process. Moreover, most of the trees, especially for large gene families, tend to be unresolved. In order to associate the rate of CNCs with gene functions, we designed a full bottom-up annotation (FBUA) pipeline to annotate the CNC variants with gene ontology (GO) categories. The FBUA annotates GO terms to the lowest levels possible (i.e. GO leaves), and therefore can provide more detailed functional information than the annotation using the fixed level of GO terms, as what most studies did [15].

Our work provides a detailed inventory of functional differences in duplicated genes that exhibit different rates of copy number changes in the ten animals, which can be used to guide future experimental or functional studies.

## Results

### Statistics of gene families

The summary statistics of gene families is shown in Table 1. Since singletons can be the result of gene loss in a gene family, we included all singleton genes and considered them as gene families of size 1. There are altogether 23,713 gene families in the 10 species. The total number of genes and gene families in most species are around 20,000 and 10,000, respectively. The number of genes per family is about 2. Comparatively, the fruitfly has the smallest number of genes and gene families, whereas the mouse has the largest number of genes and gene families. The relatively smaller number of genes and families in the fruitfly may be due to its high rate of genome-wide gene deletion [16], [17], [18], so that the fruitfly's genome may be more compact than that of the other species. Interestingly, the zebrafish has the least number of gene families but the largest average gene family size, which may be due to its frequent genome duplications [19].

**Table 1:** General statistics of gene families in 10 animal species.

Species	# of genes	# of gene families*	# of genes per family
Human	22680	12241	1.85
Macaca	21944	10395	2.11
Mouse	24118	11801	2.04
Rat	22993	10375	2.22
Cow	21755	10152	2.14
Dog	19305	9743	1.98
Opossum	19520	9267	2.11
Chicken	16736	9818	1.7
Zebrafish	21322	7766	2.75
Fruitfly	14039	9214	1.52

* includes singleton gene families.

### The relative CNC rate patterns

In order to get a distribution of the rates of CNCs, we used humans as a reference point to calculate rates of change in the other nine species because the human genome is one of the best annotated genomes and it seems intuitively more natural for people to understand changes relative to humans. Moreover, as we do not focus on determining the direction of changes, the choice of reference species should not affect our subsequent inference.

We calculated the rates of CNCs (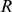) with respect to humans for all of the 23,713 gene families (see Materials and Methods for the definition of 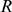), and generated rate patterns by sorting the species based on their 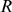 values. An example of a rate pattern is: 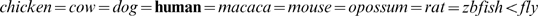. This pattern means that the fruitfly has the largest 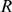 (rate of copy number changes), and *R*s of all the other species are the same. Here, we included humans (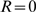) in the pattern to help determine the direction of gene family size changes (i.e. expanded or shrunk).

We clustered all gene families into groups based on their rate patterns, calling each group a *rate pattern group* (RPG). We then sorted and indexed the RPGs by descending order of the number of gene families in each group. Thus, the smaller the index of a RPG, the more gene families the RPG contains. For instance, the RPG, 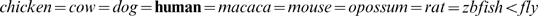, is the largest RPG containing 4,543 gene families, so it is indexed as 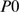. Altogether, we obtained 2,637 RPGs. Because the RPGs containing small number of gene families may be generated by noise and lack statistical power, we mainly focused on the 21 largest RPGs (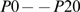) that contain no less than 100 families. These 21 RPGs contain 17,412 (73.43%) gene families, indicating that the majority of the gene families are included in these RPGs (Table 2). Based on the evolutionary scenarios inferred from these RPGs, we further classified the 21 RPGs into three categories, 1) species-specific RPGs, including P0, P1, P2, P3, P4, P5, P6, P9, P10, P11, P17, P19, and P20; 2) group-specific RPGs, including P8, P12, P13, P14, P15, and P16; and 3) conserved RPG, P7, each of which is discussed as follows.

**Table 2:** The first 21 major RPGs.

Pattern ID	Pattern content	# of gene families
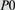	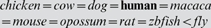	4543 (19.16%)
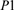	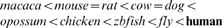	1984 (8.37%)
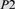	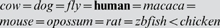	1981 (8.35%)
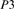	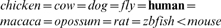	1642 (6.92%)
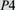	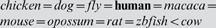	869 (3.66%)
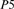	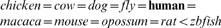	800 (3.37%)
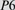	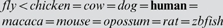	798 (3.37%)
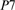	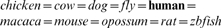	768 (3.24%)
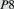	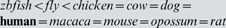	559 (2.36%)
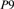	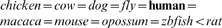	474 (2.00%)
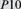	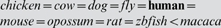	385 (1.62%)
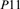	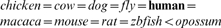	385 (1.62%)
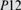	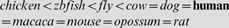	377 (1.59%)
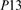	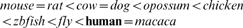	374 (1.58%)
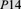	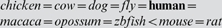	333 (1.40%)
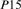	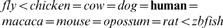	262 (1.10%)
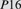	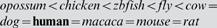	216 (0.91%)
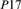	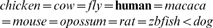	192 (0.81%)
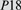	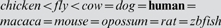	182 (0.77%)
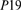	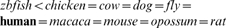	164 (0.69%)
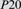	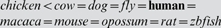	124 (0.52%)
Total		17412 (73.43%)

Sorted by the number of genes in each pattern in descending order. When species are connected with “ = ”, they are sorted by alphabetic order.

* shortened pattern IDs, which are indexed based on the number of gene families in each pattern, the smaller the ID number is the more genes the pattern has.

#### Species-specific RPGs

Species-specific RPGs are defined as the RPGs where rate changes only happened in one species. We summarized the species-specific RPGs in Table 3. There are 12 (57%) species-specific RPGs in the 21 total RPGs, suggesting that gene duplication has played an important role in species differentiation.

**Table 3:** Species-specific conjugated RPG pairs.

	RPG pairs			
	Expanded		Shrunk	
Species	Index	# of gene families	Index	# of gene families
Fruitfly	P0	4543 (49.3%)	P6	798 (8.7%)
Zebrafish	P5	800 (10.3%)	P19	164 (2.1%)
Chicken	P2	1981 (20.2%)	P20	124 (1.3%)
Opossum	P11	385 (4.2%)	P34	37 (0.40%)
Cow	P4	869 (8.6%)	P25	59 (0.58%)
Dog	P17	192 (2.0%)	P114	11 (0.11%)
Rat	P9	474 (4.6%)	P59	24 (0.23%)
Mouse	P3	1642 (13.9%)	P637	2 (0.017%)
Macaca	P10	385 (3.7%)	P78	18 (0.17%)

Within the parenthesis are the percentages of the numbers of gene families in RPGs to the total numbers of gene families in the corresponding species.

The RPGs show some interesting patterns. Specifically, we call the RPG pairs such as P0 vs. P6, P5 vs. P19, and P2 vs. P20 *conjugated* RPG pairs as the two RPGs in a conjugated pair have similar forms of rate comparison, except in opposite direction (i.e. “>” vs. “<”). We separated the conjugated RPG pairs into “expanded” and “shrunk” directions using human as reference (Table 3). Interestingly, in all the species, the number of “expanded” gene families is much larger than that of “shrunk” gene families.

Additionally, P1 is a special RPG that shows a counter-intuitive relationship between divergence time and CNC rate: the closer a species is to humans, the faster the speed of shrinkage of gene families. If we only focus on the number of CNCs, the pattern of almost all the gene families in this RPG becomes 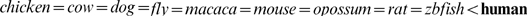. This means that in those non-human species, the CNCs in gene families belonging to P1 cannot keep up with the pace of the passage of divergence time. Based on this argument, we think P1 can be regarded as a human-specific RPG.

#### Group-specific RPGs

**Figure 1:**
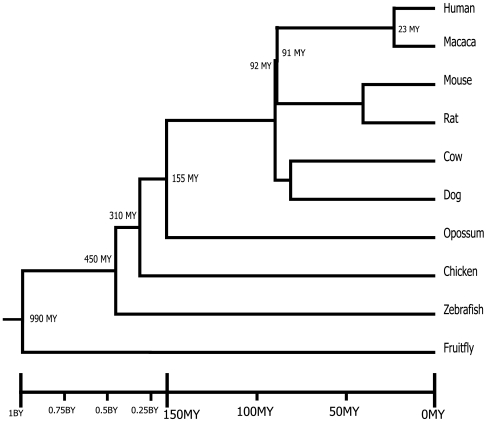
Species tree with divergence times.

Apart from species-specific RPGs, according to the species tree (Figure 1), we can also define, in the 21 RPGs, several group-specific RPGs, i.e. specific for more than one species:

(1) P8, which includes the gene families that specifically “shrunk” in zebrafish and fruitflies. Since other species are all warm-blooded animals, we took P8 as the RPG representing the difference between warm-blooded and cold-blooded animals;

(2) P12, specifically “shrunk” in chicken, zebrafish, and fruitflies, separating mammals and non-mammals;

(3) P14, specifically “expanded” in the murine lineage;

(4) P16, specifically “shrunk” in opossum, chicken, zebrafish and fruitfly, separating placentalia and non-placentalia animals.

The RPGs P15 and P18 are not as clearly defined as the above 4 RPGs due to the discrepancy between the pattens and the species tree. P15 may represent the difference between fruitflies and zebrafish.

It is interesting to show that unlike the situation in the species-specific RPGs, only P14 is an “expanded” RPG, others are all “shrunk” RPGs (excluding P15 and P18). We also found that in P8, P12, and P16, the “shrunk” speeds of the “shrunk” species, are in reverse order to their divergence time to human. For example, in P12, the pattern of “shrunk” species is 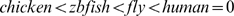. Similar to the case of P1, if the divergence time is not considered, the pattern in most of the gene families will change into 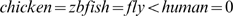. Thus, this implies that divergence time in some situations may be not a critical factor to determine the CNC difference between species or species groups. The same thing also happens in P13. If divergence time is removed, the pattern of P13 will change into 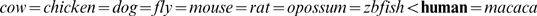. Therefore, P13 can be seen as “shrunk” RPG that separates primates and non-primates.

#### Conserved RPG

The last category of RPG contains only P7, the gene families that have equal copy numbers among all the species that we studied. There are altogether 768 families belonging to this RPG.

### GO annotation of RPGs through FBUA

It is interesting to know how the RPGs differ from each other in terms of biological functions and how functional difference is related to species difference. For this purpose, we annotated the RPGs into vectors of tip GO terms using the Full Bottom-Up Annotation (FBUA) approach that we proposed (see Materials and Methods for details). The complete annotation matrices are provided in Text S1, S2, and S3. Here we only focused on the 21 major RPGs.

There are altogether 6,964, 7,182, and 1,391 vector elements for biological processes, molecular function, and cellular component, respectively, for the 21 RPGs. Since we are mostly interested in identifying major differences between RPGs, we used the upper outlier of all elements in a RPG's vector to represent the RPG, that is, we selected the elements that are no less than 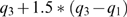 in each vector, where 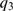 is the 75% quartile and 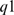 is the 25% quartile. As vector elements represent the overall probabilities that genes in a RPG are annotated to specific GO tip functions, this statistical cutoff allows us to pick out the highly enriched GO tip functions in each RPG. After removing the less frequent tips, we reassembled the remaining vectors and obtained three representative matrices that consist of 594, 355, and 165 GO tip elements for biological processes (BP), molecular functions (MF), and cellular components (CC) respectively.

To better reveal the relationships between the GO tips in the annotation matrix, we clustered the GO tips according to their similarities in the GO graphs [20]. At the same time, we also arranged the order of RPGs in the matrices based on the divergence times represented by the RPGs. Accordingly, the RPGs are ordered as
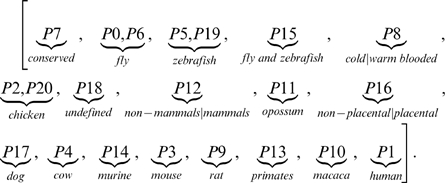


**Figure 2:**
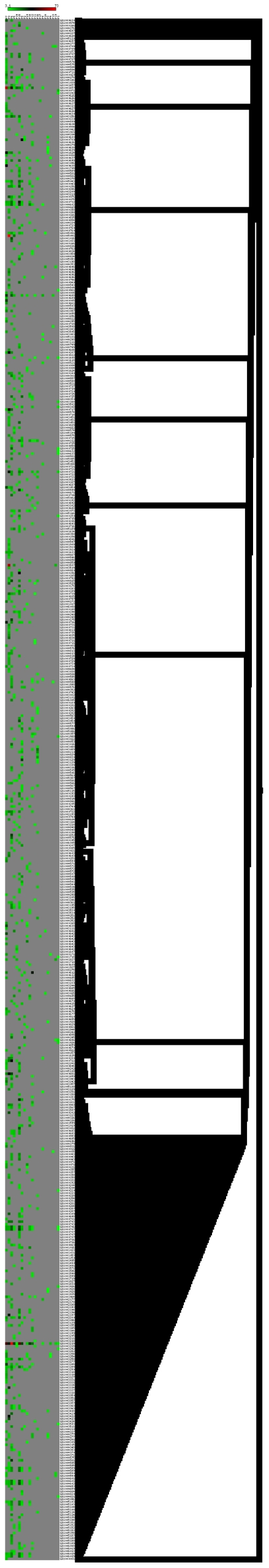
Representative annotation matrices for biological process. Grey cells in the matrix are not within the outlier of each category. The left part of the tree are generated from GO DAGs not based on the similarities of the rows of the matrix.

Figures 2–4 show the full representative annotation matrices. Gray points mean that the values of matrix elements are less than the upper outlier. We expected that signals that are located closer in the matrices are basically more similar. However, overall, this expectation is very weakly reflected in BP and MF.

**Figure 3 pone-0007342-g003:**
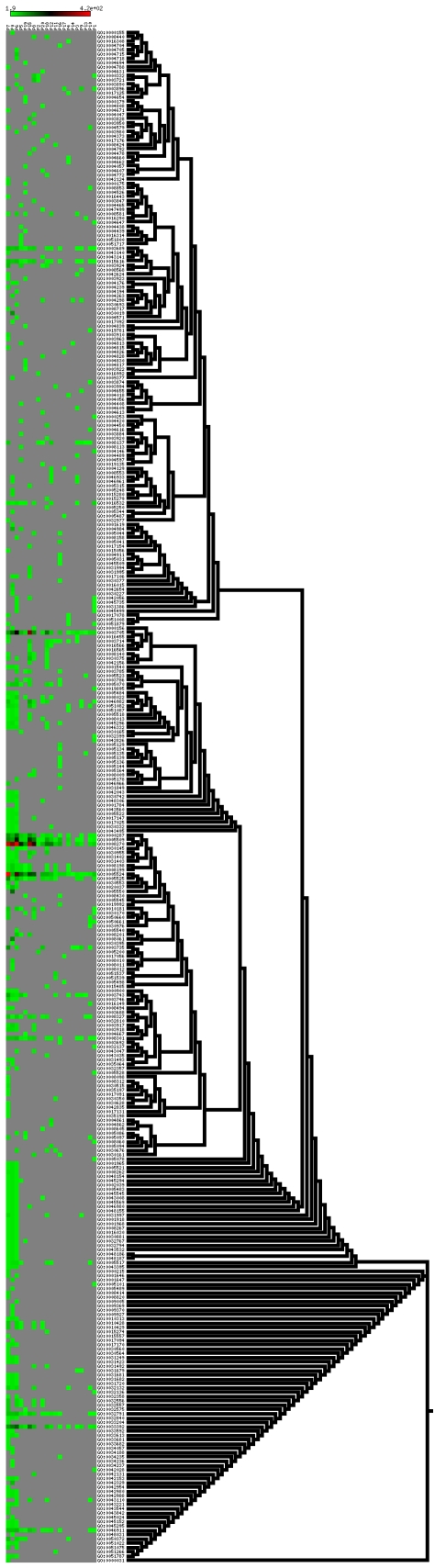
Representative annotation matrices for molecular function. Grey cells in the matrix are not within the outlier of each category. The left part of the tree are generated from GO DAGs not based on the similarities of the rows of the matrix.

**Figure 4:**
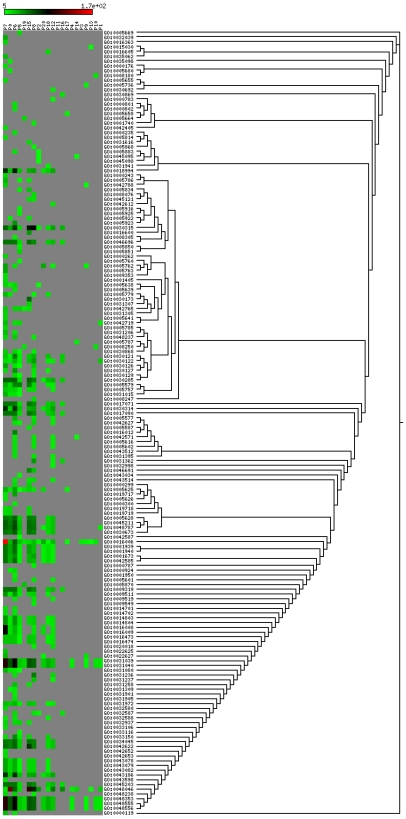
Representative annotation matrices for cellular component. Grey cells in the matrix are not within the outlier of each category. The left part of the tree are generated from GO DAGs not based on the similarities of the rows of the matrix.

Vertically, non-mammals generally tend to have more representative data points (non-gray points) than mammals do especially in CC and partially in MF and BP. RPGs that represent mammals or non-mammals are more likely to be correlated within themselves, especially in CC (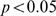, spearman's correlation) and partially in MF and BP, indicating that there might be a significant change in some aspects of gene duplication between mammals and non-mammals. In MF, P7, P0, and P6 are significantly correlated with each other (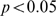, spearman's correlation), showing that conserved gene families may share a similar spectrum of functions with those fly specific gene families.

Horizontally, most of the patterns of the GO tips in BP and MF are not strictly clustered in a way that is compatible with their internal similarities in the GO graphs (showing by the trees in the left parts of Figures 2–4). But, in some local regions this expectation holds. For example, in MF, magnesium, calcium, and zinc ion binding functions (GO:0000287; GO:0005509; zinc ion binding) matched perfectly between annotation matrices and GO DAGs. Notice that some functions are highly representative for many RPGs. More than 2/3 of the RPGs have functions that are related to transcription during RNA-mediated transposition for BP; to DNA clamp loader activity, DNA translocase activity, RNA polymerase II transcription factor activity, various binding (enhancers, magnesium, calcium, zinc, ATPs, and GTPs), DNA bending activity, negative regulation of interleukin-18 production, and actin homodimerization activity for MF; and to Nebenkern, macronucleus, micronucleus, apoplast, primary endosperm nucleus, root cap mucilage biosynthetic process, and root epithelial mucilage biosynthetic process for CC. This shows that gene families with ion and ATP or GTP binding functions are more likely to undergo heterogeneous rates of CNCs in multiple species.

### Functional differences between species-specific RPGs

We summarized the highest weighted representative GO tip annotations (upper 90% quartile) in species-specific RPGs. In CC and MF, we did not find obvious species-specific differences in terms of functional categories in the top ranked GO tip annotations. In CC, almost all of the most representative GO tips are connected with nucleus, while in MF, almost all of them are related to ion binding, ATP binding or GTP binding.

In BP, the top weighted functions vary across species. About 2/3 of the differences are located in human vs. fly and human vs. zebrafish. Detailed list of functions is in Table 4. In fly vs. human RPGs (P0 and P6), we found two insect related pathways, ecdysone and chitin related pathways. But more pathways are sensory or nerve system related, such as chemical stimulus, neuropeptide signalling, corticospinal neuron axon decussation, Notch receptor processing, and sodium ion transport. We also noticed a mammalian specific pathway, alveolus development. Similar to previous studies, there are transcriptional regulatory related functions, such as the regulation of three RNA polymerase promoters, transcription anti-termination, and histone deubiquitination. RNA-mediated transposition is closely related to retroposition. There are also cancer related regulatory pathways, such as epidermal growth factor ligand processing, regulation of angiogenesis, Notch receptor processing, and Wnt signaling pathway. Maybe the most significant observation for speciation is the GO:0002077 that influences the acrosome matrix dispersal.

**Table 4:** Top species-specific annotations (BP).

Species	Pattern	Go id	Weight	GO Description
fly	P0	GO:0050911	72.98	detection of chemical stimulus involved in sensory perception of smell
		GO:0035072	62.76	ecdysone-mediated induction of salivary gland cell autophagic cell death
		GO:0007174	43.29	epidermal growth factor ligand processing
		GO:0002077	37.10	acrosome matrix dispersal
		GO:0006510	37.01	ATP-dependent proteolysis
		GO:0034223	36.86	regulation of spore wall chitin biosynthetic process
		GO:0007218	35.42	neuropeptide signaling pathway
		GO:0032199	33.93	transcription during RNA-mediated transposition
		GO:0021973	33.78	corticospinal neuron axon decussation
		GO:0033355	29.38	ascorbate glutathione cycle
		GO:0034234	27.21	regulation of spore wall chitin catabolic process
		GO:0002028	26.17	regulation of sodium ion transport
	P6	GO:0032199	72.94	transcription during RNA-mediated transposition
		GO:0016578	46.15	histone deubiquitination
		GO:0007220	37.15	Notch receptor processing
		GO:0045812	29.75	negative regulation of Wnt receptor signaling pathway, calcium modulating pathway
		GO:0016525	29.24	negative regulation of angiogenesis
		GO:0006510	29.14	ATP-dependent proteolysis
		GO:0001759	28.01	induction of an organ
		GO:0010165	26.78	response to X-ray
		GO:0048286	26.41	alveolus development
		GO:0007069	25.13	negative regulation of transcription from RNA polymerase I promoter, mitotic
		GO:0007071	25.11	negative regulation of transcription from RNA polymerase III promoter, mitotic
		GO:0006888	24.59	ER to Golgi vesicle-mediated transport
		GO:0031564	23.78	transcription antitermination
		GO:0007329	23.30	positive regulation of transcription from RNA polymerase II promoter by pheromones
		GO:0045766	23.24	positive regulation of angiogenesis
		GO:0034205	23.03	beta-amyloid formation
zebrafish	P5	GO:0032199	29.31	transcription during RNA-mediated transposition
		GO:0050911	21.01	detection of chemical stimulus involved in sensory perception of smell
		GO:0006419	19.36	alanyl-tRNA aminoacylation
		GO:0051895	17.82	negative regulation of focal adhesion formation
		GO:0031564	16.63	transcription antitermination
		GO:0046856	16.26	phosphoinositide dephosphorylation
		GO:0035072	16.12	ecdysone-mediated induction of salivary gland cell autophagic cell death
		GO:0001947	13.53	heart looping
		GO:0051898	12.87	negative regulation of protein kinase B signaling cascade
	P19	GO:0016578	23.05	histone deubiquitination
		GO:0016598	16.25	protein arginylation
		GO:0006423	12.39	cysteinyl-tRNA aminoacylation
chicken	P2	GO:0042450	19.30	arginine biosynthetic process via ornithine
		GO:0045768	12.30	positive regulation of anti-apoptosis
	P20	GO:0006434	19.97	seryl-tRNA aminoacylation
		GO:0000716	16.31	transcription-coupled nucleotide-excision repair, DNA damage recognition
		GO:0007042	12.63	lysosomal lumen acidification
		GO:0032199	10.57	transcription during RNA-mediated transposition
opossum	P11	GO:0007604	9.04	phototransduction, UV
		GO:0043576	9.02	regulation of respiratory gaseous exchange
dog	P17	GO:0006269	10.14	DNA replication, synthesis of RNA primer
cow	P4	GO:0006888	13.07	ER to Golgi vesicle-mediated transport
		GO:0021777	11.18	BMP signaling pathway involved in spinal cord association neuron specification
mouse	P3	GO:0034080	6.60	DNA replication-independent nucleosome assembly at centromere
		GO:0006335	6.51	DNA replication-dependent nucleosome assembly
rat	P9	GO:0032199	11.40	transcription during RNA-mediated transposition
macaca	P10	GO:0018279	13.18	protein amino acid N-linked glycosylation via asparagine
human	P1	GO:0000710	12.19	meiotic mismatch repair
		GO:0007218	9.05	neuropeptide signaling pathway
		GO:0043089	8.07	positive regulation of Cdc42 GTPase activity

The differences between zebrafish and human in BP are mainly focused on the transcriptional regulatory related functions and signaling pathways, such as focal adhesion formation and protein kinase B signaling cascade. There are also special pathways: heart looping and ecdysone-mediated cell death, which are also found in the case of fly vs. human comparison.

In other species vs. human cases, although the overall weights of GO tips are not high, we can still find some interesting observations. In chicken, the highest ranked difference from human is the arginine biosynthetic process via ornithine, which is closely related to the urea cycle that shows great difference between land mammals and birds. In opossum, the top representative GO function is phototransduction, which may reflect the difference of retina between opossum and human. In cow, the function of ER to Golgi vesicle-mediated transport may have something to do with milking. In human, a neuropeptide signaling pathway is among the top ranked GO tips.

### Functional differences between group-specific RPGs

Similar to the differences between species-specific RPGs, there is little difference between group-specific RPGs in CC and MF. All of the top ranked group-specific GO functions are related to nucleus in CC, while in MF most of them are related to zinc ion binding, ATP binding, and RNA polymerase II transcription factor activity-enhancer binding.

GO functions in BP differ greatly among species groups (Table 5). In fly vs. zebrafish (note that we showed that according to the pattern, P15 most likely reflects the difference between fly and zebrafish, rather than being compared with human), the most common top-ranked GO function differences are the regulation of transcription of all the three RNA polymerases (I, II and III). The retroposition related pathway is the top most difference between human and fly or zebrafish. We also observed functions that are explicitly related in phenotypic differences, such as blood vessels, neural crest cell migration, bone mineralization and bone morphogenetic protein pathway, and photoreceptor cell maintenance. The difference is also seen in regulation of some important pathways, such as transepithelial chloride transport, cell growth, and antisense RNA transcription.

**Table 5:** Top group-specific annotations (BP).

Groups	Pattern	Go id	Weight	GO Description
fly and zebrafish	P15	GO:0032199	52.20	transcription during RNA-mediated transposition
		GO:0001569	39.08	patterning of blood vessels
		GO:0007329	33.92	positive regulation of transcription from RNA polymerase II promoter by pheromones
		GO:0001755	32.47	neural crest cell migration
		GO:0007071	29.86	negative regulation of transcription from RNA polymerase III promoter, mitotic
		GO:0045494	29.42	photoreceptor cell maintenance
		GO:0046024	28.74	positive regulation of transcription from RNA polymerase III promoter, mitotic
		GO:0007069	27.89	negative regulation of transcription from RNA polymerase I promoter, mitotic
		GO:0046020	27.83	negative regulation of transcription from RNA polymerase II promoter by pheromones
		GO:0046018	26.76	positive regulation of transcription from RNA polymerase I promoter, mitotic
		GO:0030321	26.30	transepithelial chloride transport
		GO:0030502	26.04	negative regulation of bone mineralization
		GO:0001560	25.31	regulation of cell growth by extracellular stimulus
		GO:0030514	23.58	negative regulation of BMP signaling pathway
		GO:0060196	23.33	positive regulation of antisense RNA transcription
cold 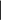 warm	P8	GO:0007004	37.74	telomere maintenance via telomerase
blooded		GO:0031295	28.86	T cell costimulation
		GO:0032199	28.40	transcription during RNA-mediated transposition
		GO:0045842	27.47	positive regulation of mitotic metaphase/anaphase transition
		GO:0007257	21.80	activation of JNK activity
		GO:0007037	18.16	vacuolar phosphate transport
		GO:0050860	17.26	negative regulation of T cell receptor signaling pathway
		GO:0045086	17.07	positive regulation of interleukin-2 biosynthetic process
		GO:0002071	16.47	glandular epithelial cell maturation
		GO:0050434	15.24	positive regulation of viral transcription
non-mammals	P12	GO:0006120	34.89	mitochondrial electron transport, NADH to ubiquinone
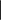 mammals		GO:0032199	22.44	transcription during RNA-mediated transposition
		GO:0016578	19.51	histone deubiquitination
		GO:0031658	13.21	G1/S-specific negative regulation of cyclin-dependent protein kinase activity
		GO:0031661	13.20	G2/M-specific negative regulation of cyclin-dependent protein kinase activity
		GO:0030317	13.03	sperm motility
		GO:0042776	11.86	mitochondrial ATP synthesis coupled proton transport
		GO:0042777	11.86	plasma membrane ATP synthesis coupled proton transport
non-placental	P16	GO:0030890	22.66	positive regulation of B cell proliferation
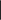 placental		GO:0042104	18.04	positive regulation of activated T cell proliferation
		GO:0048304	17.04	positive regulation of isotype switching to IgG isotypes
		GO:0042523	13.13	positive regulation of tyrosine phosphorylation of Stat5 protein
murine	P14	GO:0006465	12.00	signal peptide processing

At the demarcation of cold and warm blooded animals, most of the top ranked differences are immune system related pathways: T cell costimulation, T cell receptor signaling pathway, interleukin-2 biosynthetic process, JNK activity, and viral transcription. The next common function differences include mitosis related functions such as telomere maintenance and mitotic metaphase/anaphase transition. Retroposition related pathway is ranked the third in this group.

Between non-mammal and mammals, the big difference in rates of CNCs involves many mitochondria related functions and energy and metabolism functions. Another major functional category in this group is cyclin-dependent protein kinase activity. Here, the retroposition related pathway (GO:0032199) is also highly ranked (second) in this group.

Between non-placental and placental animals, the big difference is related to immune systems regulating T cell proliferation, B cell proliferation, and IgG isotypes. In this group, a Stat5 protein related function is also highly ranked. And in the murine lineage, only a signal peptide processing pathway has a high rank.

### Functional differences in the Conserved RPG

RPG P7 contains the gene families in which none of the species have differential rates of copy number changes. The gene families in this RPG are expected to reflect functions related to basic biological processes, so that the rates of CNCs are highly constrained by natural selection. Our results in Table 6 confirm this speculation. In BP, the highly ranked functions are related to the pathways involving histone, retroposition, RNA polymerase II, Gogi transport, splicing, snRNA, tRNA, cell cycle, docking to nuclear, and etc. Those processes are all basic and critical biological processes. In MF, the functions are similar to the highly ranked functions in other groups of RPGs.

**Table 6:** Top annotations of the conserved RPG.

Groups	Pattern	Go id	Weight	GO Description
BP	P7	GO:0016578	64.15	histone deubiquitination
		GO:0032199	47.44	transcription during RNA-mediated transposition
		GO:0006387	38.81	snRNA capping
		GO:0016245	37.80	hyperphosphorylation of RNA polymerase II
		GO:0007368	34.31	determination of left/right symmetry
		GO:0006888	30.94	ER to Golgi vesicle-mediated transport
		GO:0032858	29.67	activation of Rab GTPase
		GO:0000059	29.31	protein import into nucleus, docking
		GO:0030503	29.07	regulation of cell redox homeostasis
		GO:0006388	28.31	tRNA splicing
		GO:0006422	28.11	aspartyl-tRNA aminoacylation
		GO:0006303	25.91	double-strand break repair via nonhomologous end joining
		GO:0007095	25.68	mitotic cell cycle G2/M transition DNA damage checkpoint
MF	P7	GO:0005524	419.15	ATP binding
		GO:0008270	385.80	zinc ion binding
		GO:0005525	105.26	GTP binding
		GO:0005509	102.04	calcium ion binding
		GO:0000287	88.52	magnesium ion binding
		GO:0003735	86.97	structural constituent of ribosome
		GO:0033392	84.09	actin homodimerization activity
		GO:0003705	78.67	RNA polymerase II transcription factor activity, enhancer binding
		GO:0003743	76.45	translation initiation factor activity
		GO:0050372	55.98	ubiquitin-calmodulin ligase activity
		GO:0003924	55.34	GTPase activity
		GO:0032791	51.04	lead ion binding
		GO:0046982	49.70	protein heterodimerization activity
		GO:0046911	48.52	metal chelating activity
		GO:0003689	47.82	DNA clamp loader activity
		GO:0015616	47.82	DNA translocase activity

## Discussion

Our results show that many major RPGs are species-specific and in the direction of gene gain rather than gene loss when compared to humans. For example, in macaca, the rates of CNCs are higher than other species for 383 gene families, but lower for only 18 gene families. Even though our method cannot determine the absolute direction of CNCs, according to the most parsimonious principle, gene duplication in individual species is a more favorable explanation than gene loss in all remaining species. Since speciation is supposed to be accompanied with new genes and functions, our observations provide genome-wide evidence for this proposition. It has to be mentioned that bias and error in genome annotation seems to have a relatively small impact on our analysis. As we require the number of gene families that show the same pattern of rate comparison among species to be greater than 100, this effectively minimizes the amount of stochastic annotation error. Another factor that may influence our results is the total number of genes in the genome and it is expected that the higher the total number of genes and also the number of genes per family, the more likely that the family is observed at a high frequency. However, when one examines Table 1, it is clear that both mouse and rat genomes have the most number of genes, however, these two species have their species-specific patterns ranked not in the top, suggesting that annotation bias may not be serious.

It is hard to determine the absolute directions of CNCs in group-specific RPGs, because the parsimonious rule in this situation is not as robust as in species-specific patterns. Thus, any differences that we observed could be gene gain in one group of species, gene loss in the other, or both. However, at least, we know that variations of CNC rates in some gene families occurred between certain groups of species.

We also found that CNCs are saturated with respect to divergence time in some RPGs (P1 and P13) or some parts of RPGs (P8,P12 and P16), characterized by the fact that CNC rates are inverse to divergence time. In fact, in these cases, copy numbers do not change between specified species. This could be due to either low gene duplication rate compared to divergence time, or negative selection that prevents certain gene families from changing their copy numbers. Gene duplication rates in vertebrates have been estimated in a few studies [21], [22], and the consensus estimation is about one duplication per gene per 1000 MYs. That is to say when without selection, a gene family of 10 members will take 100 MYs to generate one new copy. Since the average family size is far less than 10, we think that rate equality of CNCs in some species is mainly due to low rates of gene duplication. On the other hand, the species that exhibit different rates of CNCs are most probably due to natural selection. For example, for P1 (human-specific) and P13 (primate group-specific), the human and macaca gene families in these two RGPs are most likely to be influenced by natural selection. It is interesting to note that P1 is the second largest RPG, implying that a much larger group of functions generated from gene duplication may have been involved in human formation than in other species.

To examine functional enrichment of the gene families that have the same comparison patterns of rates of CNCs among the species, we further annotate the RPGs using the novel FBUA. The advantage of FBUA is that it can provide much more detailed functional annotation than many “use-as-it-is” approaches where the original GO annotation is used regardless of the resolution. Because the FBUA gives the likelihood of tip GO terms, it provides a natural statistical measurement for functional enrichment in genes of interest. However, a shortcoming of the FBUA is that sometimes, it may over-annotate the functions, i.e. annotate to tip GO terms that are too deep to be applicable to the species. This problem is mainly due to the incompleteness of GO DAGs. To solve this problem, we can trace back one or two steps to get more general annotation. But for most of the time, it is not a big problem. In fact, we should not interpret the annotation results from FBUA the same as traditional methods. The tip annotation from FBUA represents the probability of the path that leads to the tip annotation from the original GO term. Therefore, we prefer to say the path leading to a certain tip rather than to emphasize the tip itself.

In general, we found that the annotations of MF and CC between RPGs show a more organized pattern than that of BP. Especially in CC, we can observe a obvious difference between mammals and non-mammals. Since CC is supposed to be a collection of conserved features in the cell, this tells us that mammals and non-mammals have undergone severe functional changes in the cell. In MF, most of the differences are between {fly, zebrafish} and other species. This does make sense because most of the differences in MF are ion-binding and ATP or GTP binding functions, which are all basic functions in organisms, so that the differences are more likely to occur between highly diverged species from human, such as fly and zebrafish. In fact, those functions are also common in gene families belonging to the conserved RPG, P7.

The most fruitful results are from BP. By comparing BP annotations between RPGs, we found many interesting functions that are associated with certain groups of species, which can find support from common knowledge or other studies. Here we just show some typical examples: 1) between fly and human, we found an insect-specific ecdysone related function that is highly ranked in P0, and we also found many nervous system related functions, which we believe are more likely to be related to human. 2) In chicken specific RPGs, the arginine biosynthetic process via ornithine was picked out by FBUA as the highest ranked function compared with other species. Since it is known that the urea cycle shows great difference between land mammals and birds, this observation indicates that this phenotypic difference may result from gene duplication. 3) we noticed that GO:0032199 (transcription during RNA-mediated transposition or retroposition related function) are simultaneously highly ranked in several groups-specific RPGs (P15, P8 and P12). In fact, the difference of retroposition activities are reported in fly [23] and zebrafish [24], relating to P15; and between mammals and non-mammals (unpublished data), relating to P8 and P12. These results prove that gene duplication plays an important role in generating specific new functions in different animals. The annotation matrix generated in our study can serve as an atlas of the rate comparison of CNCs in animals, which will help guide researchers to connect phenotypic features to certain gene duplications.

## Materials and Methods

### Collecting Data

We analyzed 10 animal genomes including human (*Homo sapiens*), macaca (*Macaca mulatta*), mouse (*Mus musculus*), rat (*Rattus norvegicus*), dog (*Canis familiaris*), cow (*Bos taurus*), opossum (*Monodelphis domestica*), chicken (*Gallus gallus*), zebrafish (*Danio rerio*), and fruitfly (*Drosophila melanogaster*). The phylogeny of these species is shown in Figure 1 (adapted from Hedges[25]). We downloaded all the gene families for the ten species from ENSEMBL [26]. We required that each gene be a protein-coding gene with no premature stop codon, more than 100 amino acids, and have a known chromosomal location. We discarded genes on mitochondria. When a species does not have a gene family, we set the copy number of the family in this species to 0.

### Calculating relative CNC rates and patterns

We used humans as the reference to calculate the CNC rate (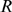) of each gene family. Specifically,
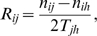
(1)where 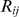 is the observed rate of family size change in family 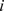 of species 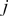 relative to the human, 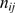 is the number of genes in family 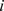 of species 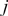 (when 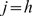, the species is human), and 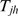 is the divergence time in million years (MY) between species 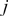 and human. The divergence times were obtained mainly from Hedges[25]. The opossum-eutheria divergence time (∼155 MY) was computed as the average of divergence time estimates in several studies [27], [28], [29]. The divergence times used in this study are shown in Figure 1. Note, the definition of 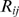 does not require any predefined models, it is the observed CNC divided by the species divergence time.

For each gene family, we generated a rate pattern by sorting the species based on their 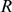 values. Here, a pattern is an ordered sequence of species according to their relative CNC rates to human. After that, we clustered the gene families of the same pattern into groups, called rate pattern groups (RPG), and thus a rate pattern group contains all the gene families that have the same rate pattern. Table 2 shows the 21 largest RPGs (i.e. containing the highest number of gene families).

### Annotating RPGs with gene ontology using FBUA

The GO terms of all the genes were extracted from Ensembl for the three categories–Biological Process (BP), Molecular Function (MF), and Cellular Component (CC). Since not all the genes have GO terms in each category and genes of the same family should perform more or less similar functions, we combined the GO terms of the genes belonging to the same gene family and removed duplicated GO terms. Then we pooled the family GO terms within each RPG. Note that, at this level, we preserved the frequencies of GO terms, which will be used as one of the parameters to determine the importance of a certain function.

The original collection of GO terms in each RPG is not suitable for representing the function spectrum of RPGs. This is because the GO terms are organized as directed acyclic graphs (DAGs) and GO terms at the same level (here level means the number of steps descending from the root) are not intrinsically comparable. To deal with this problem, for example, Karuppasamy et al.[15] annotated and compared GO terms at multiple levels. Lopez-Bigas et al.[10] simply used the GO terms that have more than 100 human genes. Both solutions are not satisfactory. In fact, Lopez-Bigas et al.[10]'s method may be significantly influenced by the systematic bias generated by the fact that certain genes tend to be better experimentally annotated than others. Also the method loses much resolution as the nodes that satisfy the criterion tend to be low level nodes (nodes that are very close to the root).

Here, we designed an annotation pipeline called Full Bottom-Up Annotation (FBUA) to deal with the aforementioned problems. We noticed that the tip GO terms (i.e. the leaves of GO graphs) contain all the information of the paths to the root. Assuming that the entire GO graphs are fully resolved, if we require that a gene with a certain GO term be fully annotated, it should be labeled as one of the tips in the current GO graphs. Therefore, we can use the tip GO terms plus their coalescent information to represent the whole DAGs. In this way, we can preserve all the information and do not have to be bothered by the bias from the selection of GO terms. However, it should be pointed out that this method can be only as good as the resolution of the entire GO graphs and carry the same bias as the GO graphs.

Formally, we assume that an internal node (GO term 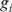) is equally likely to be annotated to any of its children, and all the paths from 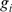 to the tip GO terms are independent of one another. The probability of the GO term 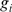 being annotated to any of the tip GO terms 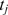, 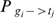, can be computed as
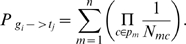
(2)

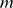
 is the path number, taking values from 1 to 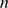, assuming that there are 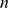 different paths going from node 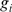 to the tip 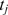, 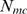 is the number of children that the parent node 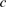 has in path 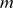, and 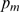 is the collection of parental nodes involved in the path 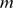 going from 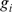 to 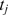, including 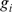.

**Figure 5:**
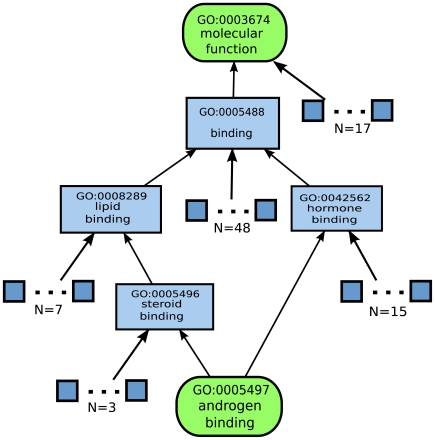
An example sub-graph of GO DAGs.

Figure 5 shows an example of how we compute the probability. The likelihood for GO:0005496 to be annotated to the tip GO:0005497 is simply 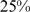 as the parent GO term has four children and is equally likely to be annotated to each one of them. The likelihood for GO:0005488 to be annotated to the tip GO:0005497 is computed as follows: there are altogether two paths from GO:0005488 to GO:0005497. The probability of GO:0005488 being annotated to GO:0005497 in path 1 is 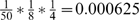, and the probability of GO:0005488 being annotated to GO:0005497 in path 2 is 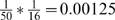. Assuming that two paths are independent, we have the final probability of GO:0005488 being annotated to GO:0005497 equal to 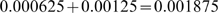.

Let 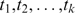 be the set of tips of the entire GO graph and 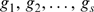 the collection of GO terms in 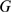. Then, 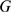 can be annotated as a vector of GO tips 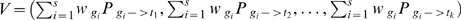, where 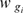 is the frequency of 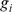 in 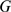 and each element in the vector represents the probability of the collection of G being annotated to a specific GO tip. Therefore, for each RPG, we can obtain an annotation vector 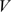.

### Clustering and plotting annotation matrix

To better reveal the relationships between the GO tips in the annotation matrix, we calculated the Jiang and Conrath's pairwise similarity distance [20] between any two GO tips using the GOSim package [30]. We then constructed Neighbor-Joining trees [31] based on the distances using the Phylip program [32]. We plotted the annotation matrix using matrix2png program [33] and combined it with the GO tip trees.

### Other data analyses

All the text parsing and processing procedures were done using a series of programs coded in OCAML language. All the statistical analyses were performed in R [34].

## Supporting Information

Text S1 biological process
(2.31 MB TXT)

Text S2 molecular function
(2.39 MB TXT)

Text S3 Cellular processes
(0.43 MB TXT)
